# Supplementary material for: Using capture‐recapture methods to estimate influenza hospitalization incidence rates
Source: Influenza Other Respir Viruses. 2021 Nov 8;16(2):308–15. doi: 10.1111/irv.12924 (PMC8818814; doi:10.1111/irv.12924)
Supplement: Supplementary file 1 — Table S1. Example of capture‐recapture estimate Table S2. Examining independence assumption for overall and sub‐populations [file IRV-16-308-s001.docx]

**Supplemental Tables**

| **Table S1. Example of capture-recapture estimate** | | | |
| --- | --- | --- | --- |
| **Research database** | **Clinical Database** | | **Total** |
|  | **Cases Clinical test** | **Cases**  **missed** |  |
| Cases enrolled | *284 (m)* | *29 (N_2_)* | *313 (n)* |
| Cases missed | *24 (N_1_)* | *2 (X)* |  |
| Total | *308 (M)* |  | *337(N)* |

| **Table S2. Examining independence assumption for overall and sub-populations** | | | | | |
| --- | --- | --- | --- | --- | --- |
| **Population** | **Probability of capture in both databases**  **(m/N)** | **Marginal Probability** | | **Product of Marginal probabilities** | **Independence Condition Satisfied*** |
|  |  | **Clinical database**  **(M/N)** | **Research database**  **(n/N)** |  |  |
| 3-year total | 0.84 | 0.91 | 0.93 | 0.85 | Yes |
| Sub-Populations |  |  |  |  |  |
| Age group |  |  |  |  |  |
| 18-49 years | 0.85 | 0.93 | 0.92 | 0.85 | Yes |
| 50-64 years | 0.82 | 0.90 | 0.91 | 0.83 | Yes |
| 65-74 years | 0.84 | 0.92 | 0.92 | 0.85 | Yes |
| 75+ | 0.87 | 0.91 | 0.96 | 0.87 | Yes |
| Race |  |  |  |  |  |
| White | 0.85 | 0.91 | 0.94 | 0.85 | Yes |
| Black | 0.83 | 0.93 | 0.90 | 0.84 | Yes |
| Sex |  |  |  |  |  |
| Female | 0.83 | 0.91 | 0.92 | 0.84 | Yes |
| Male | 0.87 | 0.93 | 0.94 | 0.87 | Yes |
| Season |  |  |  |  |  |
| 2016-2017 | 0.87 | 0.94 | 0.92 | 0.87 | Yes |
| 2017-2018 | 0.85 | 0.93 | 0.92 | 0.85 | Yes |
| 2018-2019 | 0.82 | 0.87 | 0.95 | 0.82 | Yes |
| Vaccination Status |  |  |  |  |  |
| Unvaccinated | 0.81 | 0.91 | 0.91 | 0.82 | Yes |
| Vaccinated | 0.87 | 0.92 | 0.94 | 0.87 | Yes |
| Prior Vaccination |  |  |  |  |  |
| No | 0.85 | 0.92 | 0.92 | 0.85 | Yes |
| Yes | 0.84 | 0.90 | 0.94 | 0.85 | Yes |

*****Independence condition: Probability of influenza positives captured by both databases is equal or nearly equal to the product of the marginal probabilities of influenza positives captured by clinical and research databases.
